# Summer Session of the Dental Department of the University of Maryland

**Published:** 1885-04

**Authors:** 


THE SUMMER SESSION OF THE DENTAL DEPARTMENT OF THE UNIVERSITY OF MARYLAND which began in April last, has been very successful in affording more than sufficient clinical material in the form of Infirmary patients, for the class in attendance.

The advantage of such a large amount of practical instruction which the patients who frequent this Infirmary afford, cannot be overstimated, and if the author of an editorial which appeared in a late number of the *Medical Record* would pay a visit to this institution at any period during the year, he will find that "dental charity" at which he sneers, is of a different kind from that he describes, and is of a nature to compare equally with what may be termed "medical charity." The editorial referred to as appearing in the *Medical Record* is as follows:

"DENTAL CHARITY.—Dr. Roosa struck a happy note at the dinner of the Odontological Society last week, when he urged that dentistry, being such a prosperous calling should now do a little more for charity. There is no reason why a man should receive gratuitous treatment if he has a pain in the stomach, but none if he has a pain in his tooth. He can, to be sure, have his tooth pulled out without paying, but that is about the limit to which dental charity has as yet reached. The dental departments of our city dispensaries consist generally of a box of second-hand forceps, which are wielded by young amateurs with a generous indiscrimination, and with varying degrees of skill. Such kind of dental charity may be compared to a surgical charity, which is supplied simply with a saw and a knife for cutting off all bruised or injured fingers, without making any finical distinctions as to degree or kind of traumatism. There are annually in this city not far from ten thousand teeth wrenched from the poor man's mouth in the name of charity. How much discomfort, dyspepsia, and domestic troubles this produces we will not attempt to estimate. Certain it is that the teeth of the poor get a great deal less care than they ought to have, and are more abused, surgically speaking, than any other organ of the body. In the name of charity, let the poor be taught how to preserve their teeth, and let the clean mouths, at least, be given a chance to have their good members saved."

